# Alert sign and symptoms for the early diagnosis of pulmonary tuberculosis: analysis of patients followed by a tertiary pediatric hospital

**DOI:** 10.1186/s13052-022-01288-5

**Published:** 2022-06-13

**Authors:** Elisa Farina, Carmen D’Amore, Laura Lancella, Elena Boccuzzi, Marta Luisa Ciofi degli Atti, Antonino Reale, Paolo Rossi, Alberto Villani, Massimiliano Raponi, Umberto Raucci

**Affiliations:** 1 Unit of Internal Medicine, Celio Military Hospital, Rome, Italy; 2 Clinical Pathways and Epidemiology Unit, Bambino Gesù Children’s Hospital, IRCCS, Rome, Italy; 3 Division of Immunology and Infectious Diseases, Department (DPUO), University-Hospital Pediatric, Bambino Gesù Children’s Hospital, IRCSS, Rome, Italy; 4 Department of Emergency and Clinical Pediatrics, Bambino Gesù Children’s Hospital IRCCS, Rome, Italy; 5 Medical Direction, Bambino Gesù Children’s Hospital, IRCSS, Rome, Italy

**Keywords:** Children, Tuberculosis, Early diagnosis, Emergency

## Abstract

**Background:**

Intercepting earlier suspected TB (Tuberculosis) cases clinically is necessary to reduce TB incidence, so we described signs and symptoms of retrospective cases of pulmonary TB and tried to evaluate which could be early warning signs.

**Methods:**

We conducted a retrospective descriptive study of pulmonary TB cases in children in years 2005–2017; in years 2018–2020 we conducted a cohort prospective study enrolling patients < 18 years accessed to Emergency Department (ED) with signs/symptoms suggestive of pulmonary TB.

**Results:**

In the retrospective analysis, 226 patients with pulmonary TB were studied. The most frequently described items were contact history (53.5%) and having parents from countries at risk (60.2%). Cough was referred in 49.5% of patients at onset, fever in 46%; these symptoms were persistent (lasting ≥ 10 days) in about 20%. Lymphadenopathy is described in 15.9%. The prospective study enrolled 85 patients of whom 14 (16.5%) were confirmed to be TB patients and 71 (83.5%) were non-TB cases. Lymphadenopathy and contact history were the most correlated variables. Fever and cough lasting ≥ 10 days were less frequently described in TB cases compared to non-TB patients (*p* < 0.05).

**Conclusions:**

In low TB endemic countries, pulmonary TB at onset is characterized by different symptoms, i.e. persistent fever and cough are less described, while more relevant are contact history and lymphadenopathy. It was not possible to create a score because signs/symptoms usually suggestive of pulmonary TB (considered in the questionnaire) were not significant risk factors in our reality, a low TB country.

**Supplementary Information:**

The online version contains supplementary material available at 10.1186/s13052-022-01288-5.

## Background

Tuberculosis (TB) is one of the top 10 causes of death worldwide [1]**.** It typically affects lungs (80% are pulmonary TB) but can also affect other sites (extrapulmonary TB 15–20%). In order to contain epidemic and achieve the END TB strategy (TB elimination), defined by WHO [2] new strategies are needed, such as screening programs, contact tracing [3–5]**,** early detection of suspected cases and preventive therapy to reduce disease progression in infected patients (treating latent TB) [6, 7]**.** Childhood disease is often acquired following close contact with an infectious adult case, and in 30–40% involves household contacts [8, 9]. The risk of progression is higher (40–50%) in children < 5 years especially in the first 12 months after infection [10, 11], and severe TB disease, including TB meningitis, is highest in infants (< 2 years) and usually occurs within few months of primary infection [9, 12]**.** Adult risk of progression is lower, only 5% of patients infected develop active disease. Diagnosis of TB in children is often challenging because it’s paucisyntomatic at onset; gold standard in diagnosis remains culture of bacteria, but as it can take up to 8 weeks, and considering the paucibacillary nature of childhood lung disease, microbiological confirmation is possible in 35–40% of children [13]. In these cases diagnosis is based on the triad: clinical findings, risk factors and radiographic pattern (Table 1) [14–18].

**Table 1:** Characteristic triad for the diagnosis of tubercolosis

1) Hystory of recent contact with Tubercolosis	
2) Positive Mantoux and/or IGRA (Interferon Gamma Release Assay)	
3) Signs and symptoms typical of TB and/or. Suggestive radiologic patterns	persistent fever, unremitting cough, night sweats, fatigue, weight loss, hemoptysis, chest pain consolidations, pleural effusion, cavitation, miliary pattern, hilar adenopathy with or without airway compression

The most frequently reported symptoms in literature are unremitting cough (≥ 2 weeks duration) and persistent fever (> 2–3 weeks) [4, 19–22]**.** Considering that most studies have been conducted in high prevalence countries [19, 23–26], we analyzed children’s signs/symptoms, risk factors and anamnestic data of TB at onset in a low prevalence setting. So far several attempts have been made to validate clinical scores for the early diagnosis of pulmonary TB [24, 27–29], but none of them have been internationally validated and in most cases without a control group [30]. The aim of the study was to describe signs and symptoms of retrospective cases of patients with pulmonary TB and to evaluate alerts that could intercept earlier suspected TB cases in emergency setting [31, 32].

## Methods

The study was conducted at the Bambino Gesù Pediatric IRCCS in Rome, a tertiary urban pediatric hospital. The study was approved by the Ethics Committee of our Children’s Hospital according to the Declaration of Helsinki.

### Retrospective study

The retrospective study included children ≤ 18 years through the electronic health records with diagnosis of pulmonary TB between 2005–2017 years, admitted to Hospital. The extracted data included age, sex, signs and symptoms at onset, risk factors, anamnestic data, diagnostic laboratory and imaging exams. Analyzing literature and our center’s experience we created a questionnaire for a prospective study based on TB clinical presentation and risk factors, in order to create a clinical score.

### Prospective study

We analyzed patients conducted in the emergency department (ED) between 2018–2020 with symptoms suggestive of pulmonary TB. Children < 18 years of age who arrived in the ED with one or more of the following inclusion criteria were recruited: Fever lasting ≥ 10 days; Cough lasting ≥ 10 days; Idiopathic weight loss in the past 3 months; Positive Mantoux and/or Interferon gamma release assay (IGRA/Quantiferon); Chest X-Ray suggestive for TB; History of contact with TB patient. Once recruited a questionnaire was administered to parents, assessing signs/symptoms and anamnestic data, and a written consent was obtained. Exclusion criteria were represented by chronic lung disease (like cystic fibrosis, patients with tracheostomy), infectious diseases (like Chickenpox, measles) or not giving written consent. Patients were followed up to the diagnostic definition (TB or other diagnosis) by monitoring the data obtained from the electronic health records. Mantoux was read after 48–72 h after injection and considered positive according to national guidelines [20]**.**

### Statistical analysis

Patients were characterized according to demographic and clinical characteristics. We compared signs and symptoms of TB patients identified in the retrospective studies with those of the prospective one; moreover, TB-patients and non-TB patients were compared in the prospective study. Results were summarized as numbers and percentages (categorical variables) and as mean and standard deviation (SD)(continuous variables). Categorical variables were compared with χ2 or Fisher’s 2-tailed exact test in a contingency table *r* x *c*; a Fisher test was used when the value in any of the cells of the contingency table was below five. Continuous variables were compared with a Student’s t-test. Unadjusted odds ratios (ORs) and confidence intervals (CIs) were computed through logistic regression modelling. A forward stepwise logistic regression analysis was used to determine the effect of independent risk factors for TB; those variables with p less than 0.20 were included in the multivariate analysis. Sensitivity, specificity, positive predictive value and negative predictive value were calculated for the identified risk factors. The statistical analysis was performed with STATA, statistical software version 13 (StataCorp, College Station, TX, USA).

## Results

### Retrospective Study

The retrospective study included 226 pediatric patients with a final diagnosis of pulmonary TB between 2005–2017. Mean age was about 5.6 years (± 5.2), most frequently male (52.2%, Table 2).

**Table 2:** Demographic characteristics, risk factors and reported symptoms at onset in the retrospective population group

VARIABLES	*N* = 226	%
Mean age (years) ± standard deviation (SD)	5.6 ± 5.2	
Sex		
M	118	52.2
F	108	47.8
RISK FACTORS/ANAMNESTIC DATA		
History of Contact	121	53.5
Travel in high risk countries	11	4.9
Born in high risk country	29	12.8
Parents origin’s from high risk countries	136	60.2
Immunosuppression or immunosuppressive therapy	4	1.8
Antibiotic therapy	92	40.7
- With no benefit	91	98.9
Positive Quantiferon (analyzed before hospitalization)	37	16.4
Positive Mantoux (analyzed before hospitalization)	94	41.6
SIGNS/SYMPTOMS		
Cough	112	49.6
Cough lasting ≥ 10 days	49	21.7
Fever	104	46.0
Fever lasting ≥ 10 days	44	19.4
Lymphadenopathy	36	15.9
Asthenia	35	15.5
Weight loss	22	9.7
Chest pain	16	7.1
Hemoptysis	6	2.6
Night sweats	4	1.8
DIAGNOSTIC TOOLS		
Mantoux: positive ( +) (≥ 5 mm)	210	92.9
QTF +	216	95.6
Culture +	86	42.2
PCR +	79	37.6
Bacterioscopic +	40	18.0

*QTF* Quantiferon, *PCR* Polymerase Chain Reaction

Parents came from high risk countries in 136 cases (60.2%); contact history was referred in 121 patients (53.5%). Antibiotic therapy has been administered to 40.7% (*n* = 92) of patients, and 98.9% (*n* = 91) of them referred no benefit. Patients with positive Mantoux and/or IGRA before admission were 41.6% (*n* = 94) and 16.4% (*n* = 37), respectively; 29 patients (12.8%) were born in high-risk countries and 11 had history of travel to high-risk countries (4.9%). Few patients were immunosuppressed or received immunosuppressive therapy (*n* = 4; 1.8%).

Cough was the most reported symptom at onset in 49.6% (*n* = 112) of cases; persistent cough lasting ≥ 10 days was referred in 21.7% (*n* = 49) of patients (Table 2). Fever occurred in 46% of patients (*n* = 104) and in 19.4% of patients (*n* = 44) it lasted ≥ 10 days. Other less frequently reported symptoms were lymphadenopathy (*n* = 36; 15.9%), asthenia (*n* = 35; 15.5%), weight loss (*n* = 22; 9.7%), chest pain (*n* = 16; 7.1%), hemoptysis (*n* = 6; 2.6%) and night sweats (*n* = 4; 1.8%).

The diagnostic tools used in our retrospective patients were IGRA, Mantoux and microbiological tests (culture, bacterioscopic and PCR). The latter were performed by collecting 3 gastric aspirate samples on 3 consecutive days. Mantoux and IGRA were positive in 92.9% (*n* = 210) and 95.6% (*n* = 216) of cases, respectively; 86 patients (42.2%) had positive results to microbiological cultures, 79 (37.6%) had positive PCR and 40 (18.0%) had positive results to bacterioscopic.

### Prospective study

The prospective study includes 85 patients including 14 with TB (16.5%) and 71 non-TB patients (83.5%) (Table 3). Patients with TB were older than non-TB patients (9.6 ± 5.7 *versus* 5.9 ± 4.5, respectively; *p* = 0.01); no significant differences between the two groups were observed by sex. Persistent cough lasting ≥ 10 days, is more frequently reported in non-TB patients (67.6% *versus* 21.4% of TB patients, *p* = 0.002;). Chest pain was described in 33.3% of TB patients with cough, compared to 22.9% of non-TB cases (*p* = 0.6); hemoptysis is described in 1 non-TB patient.

**Table 3:** Demographic characteristics, risk factors and reported symptoms at onset of the prospective study

Variables in the Prospective study	TB cases *N* = 14	%	Non TB cases *N* = 71	%	*P* value
Sex					
Male	5	35.7	39	54.9	0.2
Female	9	64.3	32	45.1	
Age (in years) Mean ± (SD)	9.6 ± 5.7		5.9 ± 4.5		0.01
RISK FACTORS/ANAMNESTIC DATA/SYMPTOMS					
Fever ≥ 10 days	1	7.1	29	40.8	0.02
Cough ≥ 10 days	3	21.4	48	67.6	0.002
-chest pain	1	33.3	11	22.9	0.6
-hemoptysis	0	0.0	1	2.1	0.9
Asthenia	7	50.0	43	60.0	0.5
Weight loss	3	21.4	14	19.7	0.9
Lymphadenopathy	6	42.9	11	15.5	0.03
Night sweats	0	0.0	24	33.8	0.008
History of contact	7	50.0	5	7.0	< 0.001
Travel in high risk countries	1	7.1	7	9.9	0.8
Antibiotic therapy	3	21.4	49	69.0	0.002
- no benefit	3	100	38	77.5	0.3
Born in high risk country	2	14.3	5	7.1	0.4
Immunosuppression or immunosuppressive therapy	0	0.0	0	0.0	-

*TB* tubercolosis, *SD* Standard Deviation

Fever lasting ≥ 10 days was reported in 40.8% of non-TB patients compared to 7.1% of TB patients (*p* = 0.02). Lymphadenopathy was most frequently reported in TB patients (*n* = 6; 42.9%), compared to 15.5% (*n* = 11) of non-TB cases (*p* = 0.03). Night sweats were reported in 33.8% (*n* = 24) of non-TB patients (p = 0.008). Antibiotic therapy was administered to 69% of non-TB patients (*n* = 49) and to 21.4% (*n* = 3) of TB patients (*p* = 0.002); patients receiving antibiotic therapy without improvement were 77.5% (*n* = 38) in non-TB patients and 100% (*n* = 3) in TB patients (*p* = 0.3). Contact history was reported in 50% of patients with TB and in 7% of non-TB cases (*p* < 0.001). No statistical differences between the two groups were observed for other anamnestic factors as: born in high risk countries (14.3% TB cases *versus* 7.1% non-TB cases, *p* = 0.4), travel in high risk countries (7.1% TB cases *versus* 9.9% non-TB cases, *p* = 0.8), fatigue/asthenia (50% TB cases *versus* 60% non-TB cases, *p* = 0.5) and weight loss (21.4% TB cases *versus* 19.7% non-TB cases, *p* = 0.9). None of the included patients was immunosuppressed or received immunosuppressive therapy.

Persistent cough lasting ≥ 10 days was present in 21.7% of TB patients analyzed retrospectively and in 21.4% of TB cases in the prospective study; persistent fever lasting ≥ 10 days was present in 19.0% of retrospective cases and 7.1% of prospective cases (Table 4).

**Table 4:** Duration of cough and fever, comparison between retrospective and prospective studies Retrospective study

Cough	Cases in which duration is specified	Mean duration in days (range min–max)	Min	Max	Prevalence of cases with symptom lasting ≥ 10 days
Days	84	20 (1–171)	1	171	49 (21.7%)
Fever					
Days	92	12 (1–90)	1	90	43 (19.0%)
Prospective study					
Variable		**TB cases**	**Cases with symtpom lasting ≥ 10 days**		
N° patients		14			
With Cough			3 (21.4%)		
With Fever			1 (7.1%)		

*TB* tubercolosis

The multivariate analysis identified history of contact and lymphadenopathy as risk factors for pulmonary TB (Table 5).

**Table 5:** Risk factors for pulmonary tubercolosis: multivariate analysis

Variables prospective study	Crude OR (IC 95%)	Adjusted ^a^ OR (IC95%)
Fever ≥ 10 days	0.11 (0.01–0.9)	0.03 (0.001–1.0)
Cough ≥ 10 days	0.13 (0.03–0.5)	0.05 (0.003–0.7)
Lymphoadenopathy	4.1 (1.2–14.1)	28.6 (2.1–391.2)
History of contact	13.2 (3.3–52.8)	19.8 (1.4–287.0)
Weight loss	1.1 (0.3–4.5)	6.9 (0.4–117.5)

^a^ adjusted for fever ≥ 10 days, cough ≥ 10 days, lymphadenopathy, history of contact and weight loss

## Discussion

TB diagnosis in children is often delayed because clinical presentation at onset is non-specific and there isn’t always a history of contact/exposure to a TB case. Furthermore, diagnostic methods available often don’t give immediate confirmation [33]. The two cohorts analyzed (retrospective and prospective) allowed us to extrapolate signs/symptoms at onset and anamnestic data (including risk factors), in order to evaluate whether there’s a different clinical presentation and/or different risk factors in low a prevalence country compared to what is described in literature. In the prospective study a questionnaire was administered to parents, assessing clinical symptoms and anamnestic data. So far most studies have been conducted in high TB incidence countries [19, 23–26, 34]. In these countries, the correlation between respiratory symptoms and TB may be different than in low endemic countries. In addition, risk factors may also have a different weight, for example contact with a TB case may be a less discriminating factor as frequently present in anamnesis, meanwhile in low-endemic countries it assumes a greater weight.

Contact is one of the most significantly correlated risk factor, described in 53.5% of patients with TB in the retrospective study and in 50% of cases in the prospective study, while only 7% among non-TB patients (prospective study). The OR (adjusted 19.8) confirms the correlation between contact and TB, as in other studies where contact is a frequently described risk factor (53.2–65.2% of patients) [20, 29, 35]. Therefore this data will certainly have a relevant weight in a clinical score. Unfortunately, a child is often just a sentinel case of an adult infection [36].

Another important risk factor is having parents from countries at risk, as highlighted in our retrospective series in 60.2% of cases and confirmed in other studies [20, 37, 38]. In the prospective study parents didn’t declare on the questionnaire their citizenship/native country, so we don’t have this data (question to add to the questionnaire proposed).

Being born in countries at risk is infrequent in our cohorts (12.8% of retrospective cases, 14.3% of prospective). This obviously depends on the country in which the study is performed, for example in USA a higher percentage is described (31% of children with TB are born abroad) [38].

Another variable investigated is use of antibiotic therapy and whether with or no benefit. In our study almost all of our TB patients treated previously with first-line antibiotics had no benefit: in the retrospective cohort 91/92 (98.9%) referred no benefit; in the prospective study 3/3 (100%). Comparing the prospective TB and non-TB cases (Table 4), antibiotic therapy was mostly prescribed in non-TB cases (69% vs 21.4%) and this probably correlates with higher frequency in this group of fever and persistent cough, and consequently prescription of first-line therapy. In the non-TB group 77.5% of children referred poor response to antibiotic therapy, not so striking as in the TB patients, but expected because if they improved they wouldn’t have come to the ED. To date various studies identified poor response to first-line therapy as an element of suspicion [4, 20, 28, 39, 40]**,** however a large-scale comparison between positive and negative cases is not reported, but necessary to confirm these findings.

Immunosuppression or the intake of immunosuppressive drugs is not significant in our cohorts, in which there is a low incidence of HIV.

Focusing on symptoms at onset, in low income countries patients often present to healthcare facilities in advanced stages of the disease [41], while in high income countries physicians are questioned almost immediately, so diagnosis is often made in a subclinical phase, before symptoms becoming persistent. A symptom frequently reported in literature as suggestive and associated with TB is cough, considered of strong suspicion especially if lasting > 2 weeks(15–17) and described in 44–52% of children at onset [42–44]. Unlike what is described in international literature, in our retrospective population 49.6% of cases referred cough, but persistent only in 21.7%, and the same result emerged among TB-cases of the prospective study with persistent cough in 21.4% of patients, similarly to previous Italian studies (28.7%) [20].

Another symptom referred is fever which occurs in 46% of retrospective cases, but lasting ≥ 10 days in only 19%, while there are few cases with persistent fever at onset in the prospective cohort (7.1%). These data differs from the 35–47% frequency described in literature, both in italian studies/low prevalence countries [20, 29], and internationally [22, 45]. Comparing duration of cough and fever in the two cohorts (retrospective and prospective, Tables 3, 4 and 5), although with different proportions of patients analyzed, it seems that persistent cough and fever (lasting ≥ 10 days) is not so strongly suggestive of TB diagnosis in our Italian reality. The prospective study, comparing cases with and without TB, allowed us to evaluate the correlation between fever and persistent cough with the diagnosis of TB, and it seems to confirm this poor correlation. In the multivariate analysis the OR with a value of 0.03 for fever ≥ 10 days, and 0.05 for cough ≥ 10 days shows an almost protective effect for TB, so they seem more related to other diagnoses. These findings can have 2 different explanations: on one hand, diagnosis is often made in a subclinical phase, and this allows treatment in early stages of the disease; on the other hand, the low prevalence determines a lower probability that a child with a persistent cough and fever will have TB in our reality.

Another variable analyzed is lymphadenopathy, which is not always a symptom that lead to suspect TB, but if persistent despite first-line therapies and isolated symptom, needs to exclude Non-Tuberculous Mycobacterium infection. In clinical scores proposed for the diagnosis of TB lymphadenopathy is usually included, and frequency at onset is described (19.8–23.8%) [29, 46]. In our retrospective analysis 15.9% of cases had lymphnode involvement at onset, while in the prospective cohort lymphadenopathy was referred in 42.9% of TB cases compared to 15.5% of non-TB, with an OR of 28.6 (95%CI 2.1–391.2), so probably it’s a symptom that could have a greater weight in our population and is likely underestimated. The very large CI95% is related to the low sample size and less accurate estimate.

Weight loss, which international studies reported in 22.6–27% of cases [29, 42], was reported in 9.7% of patients in the retrospective analysis, similarly to what is described in low prevalence countries like Italy (9.9% of cases) [20]**.** The prospective analysis, on the other hand, highlights a higher frequency (21.4% in TB cases, 19.7% non-TB cases). However it’s not a discriminating factor for diagnosis, therefore in our screening process it won’t have a significant weight.

Another symptom usually associated with TB is asthenia, in some studies in 36.6% of patients [29, 41], while from our retrospective cohort it seems less frequent(15.5% of cases). In the prospective study instead it’s equally described both in patients with (50%) and without (60%) TB, therefore it doesn’t discriminate positive patients from negative ones. This higher frequency may be related to the questionnaire submitted to parents, which points out a less alarming symptom compared to others (such as fever, cough, lymphadenopathy).

Chest pain is another symptom typically associated with pleuritic involvement [17], mostly described in older children, as the ability to express this symptom is different [19]. Our retrospective case study with 7.1% of cases confirms the incidence described in the national studies (5%) [20]. In the prospective study 1/14 (7%) TB patients presented chest pain at onset, 11/71 (15%) non TB patients, with no statistically difference. However, in the questionnaire it was associated with cough symptom, but as these symptoms aren’t always related, it will be better to separate them (proposed questionnaire in Additional File 1).

Hemoptysis and night sweats are rarely reported symptoms in our retrospective cohort (2.6% and 1.8% of cases respectively), similarly to other italian studies (haemoptysis 2.7%, night sweats 1% cases) [20] and international ones [10, 47]. In the prospective cohort night sweats are never described in TB cases, and described in 33.8% of non-TB cases, probably linked to fever symptom. Therefore, these variables are not clinically relevant for early diagnosis.

We finally analyzed diagnostic tests performed, considering retrospective data (larger sample with 226 patients). Both Mantoux and IGRA were positive in most patients (92.9% and 95.6% respectively), while only 42.2% were culture positive. Although culture is gold standard for active TB diagnosis, it’s lack of positivity must not delay the diagnostic-therapeutic process, considering children’s higher risk of progression to disease after infection [7, 8, 29, 48–51]. Low sensitivity and specificity of microbiological tests is related to the paucibacillary nature of pediatric TB, with only 20–50% of pulmonary TB cases being culture positive [52]. Bacterioscopic test was positive only in 18% of cases, compatible with it’s low described sensitivity (60%)(45), while PCR was positive in 37.6% of our patients, similarly to other studies reports (30.4–32%) [20, 35].

## Conclusion

Our study highlighted that in our country, at low TB prevalence, pulmonary TB has a different clinical pattern. Persistent cough and fever are less frequently reported in our patients, while significant risk factors pointed out are contact history with TB case and lymphadenopathy. Another element of suspicion, although not statistically significant in our study because of small sample size, is poor response to antibiotic therapy in TB patients. Anamnestic data like child’s and parents’ origins are also crucial. In order to validate a clinical score for pulmonary TB in a low prevalence country like Italy, it’s necessary to review risk factors that must guide the diagnostic-therapeutic workup. In our population with low HIV and TB prevalence, the most correct inclusion criteria could be the following: Fever lasting ≥ 10 days; Cough lasting ≥ 10 days; Poor response to antibiotic therapy; Lymphadenopathy; Mantoux and/or IGRA positive; Chest X-ray suspected for TB; History of contact with TB case.

It’s necessary to increase sample involving more Centers in order to validate a clinical score that allows a promptly diagnosis, limiting the number of diagnostic tests where unnecessary**.** From this preliminary analysis, its feasibility emerged, albeit with some variations both in terms of the inclusion criteria, and on the questionnaire (illustrated in Additional File 1).

Finally, only the comparison between cases with and without TB on a larger sample will be able to confirm or highlight the red flags that must lead us to suspect TB in order to avoid unnecessary risks of interpersonal transmission, particularly in overcrowded places like ED waiting rooms.

## Supplementary Information

**Additional file 1: **Anamnestic questionnaire proposed in our study.

## Data Availability

The datasets generated and analysed during the current study are not publicly available due privacy protection but are available from the corresponding author on reasonable request and subject to the permission being obtained from Ospedale Pediatrico Bambino Gesù, IRCCS, Rome.

## Abbreviations

ED: Emergency Department
IGRA: Interferon-gamma release assay
IRCCS: Istituto di Ricovero e Cura a Carattere Scientifico (Scientific Institute for Research, Hospitalization and Healthcare)
TB: Tuberculosis
