# Supplementary material for: Alert sign and symptoms for the early diagnosis of pulmonary tuberculosis: analysis of patients followed by a tertiary pediatric hospital
Source: Ital J Pediatr. 2022 Jun 13;48:90. doi: 10.1186/s13052-022-01288-5 (PMC9195307; doi:10.1186/s13052-022-01288-5)
Supplement: Supplementary file 1 — Additional file 1: Anamnestic questionnaire proposed in our study. [file 13052_2022_1288_MOESM1_ESM.doc]

ANNEX A. Anamnestic questionnaire proposed in our study

| **Symptoms** | Yes | No |
| --- | --- | --- |
| Does the baby have cough? |  |  |
| If so, for more than 10 days? |  |  |
| If so, have you ever noticed blood with cough? |  |  |
| Has he/she ever complained for chest pain?* |  |  |
| Does the child appear more tired? |  |  |
| Has he/she lost weight or slowed growth over the last 3 months? |  |  |
| Has he/she ever had night sweats? |  |  |
| Does he/she have fever or low-grade fever (ie temperature >37.2°C)? |  |  |
| If so for more than 10 days?* |  |  |
| Specify duration* |  | |
| Did he/she take antibiotic therapy in the last month? |  |  |
| If so, have you noticed any improvement? |  |  |
| In the last 3 months have you ever noticed an increase in the size of the lymph nodes? |  |  |
| If so specify location (neck, groin, axillary, behind the ear ..)* |  | |

| **Other family news and tests performed:** | Yes | No |
| --- | --- | --- |
| Has your child done a chest x-ray in the last 6 months?  (If so, show the report during the visit) |  |  |
| Has he/she been vaccinated for tubercolosis (BCG)? |  |  |
| Has he/she ever done tuberculin skin test (Mantoux)? |  |  |
| If so, was the result positive? |  |  |
| Are there relatives with positive tuberculin skin test (Mantoux)? |  |  |
| Has he/she been in contact with someone with Tuberculosis?  If so, specify if the person affected was: |  |  |
| - a family member |  |  |
| - Cohabitant |  |  |
| - Classmate |  |  |
| - Game/sport mate |  |  |
| - other,  specify |  |  |
|  | |
| Was the child born in Italy? |  |  |
| If not specify the country were he/she was born* |  | |
| Has the child been born or stayed in the last 6 months in one of the following countries: India, Indonesia, Africa, South America, Pakistan, China, Philippines, Nigeria, South East Asia? |  |  |
| Are parents Italians?* |  |  |
| If not, specify native country * |  | |
| Is the child immunodepressed? (takes immunosuppressive drugs, is HIV positive, takes chronically corticosteroids, has an immune deficiency, etc.) |  |  |

* modified item respect to previous utilized model
